# Interstrain recombinants of human cytomegalovirus uncouple glycoprotein display, virion infectivity, and spread characteristics

**DOI:** 10.1128/jvi.01592-25

**Published:** 2025-12-30

**Authors:** Christopher S. Peterson, Ian T. Bailey, Jean-Marc Lanchy, Ivan Gallego, Brent J. Ryckman

**Affiliations:** 1 Division of Biological and Biomedical Sciences, University of Montana 205252, Missoula, Montana, USA; 2 Department of Molecular Medicine, Mayo Clinic 314206 , Rochester, Minnesota, USA; 3 Center for Biomolecular Structure and Dynamics, University of Montana 307078, Missoula, Montana, USA; Dartmouth College Geisel School of Medicine, Hanover, New Hampshire, USA

**Keywords:** phenotypic variation, genetic diversity, cytomegalovirus

## Abstract

A prevailing model holds that human cytomegalovirus (HCMV) spreads cell-to-cell spread upon initial culturing from clinical specimens and that *de novo* mutations in the UL128-131 genes reduce expression of glycoprotein complex gH/gL/UL128-131 (pentamer) relative to gH/gL/gO (trimer), which in turn enhances virion infectivity and favors cell-free spread. The clone Merlin-BAC (ME) expresses far more pentamer than trimer and is highly cell-associated, whereas clone TB40-BAC4 (TB) expresses mostly trimer and spreads predominantly cell-free. A single G>T polymorphism in TB relative to ME was shown to impair UL128 mRNA splicing, reducing pentamer expression and enhancing infectivity and cell-free spread. However, enhanced cell-free spread of ME due to pentamer suppression did not come at the expense of efficient cell-to-cell spread, and cell-to-cell spread of TB was especially poor despite highly infectious intracellular virus. Most of the nucleotide diversity in the HCMV genome is due to 17 genes that have up to 14 alleles each, and TB and ME match at only two of the 17. Here, we report a set of recombinant HCMV generated by coinfecting cells with TB and ME. Trimer:pentamer ratio and virion infectivity largely aligned with the TB or ME UL128, with “TB-like” recombinants displaying more trimer and higher infectivity and “ME-like” recombinants displaying more pentamer and lower infectivity. Strikingly, some recombinants had shifts in preference for cell-free vs cell-to-cell spread without predicted changes to trimer:pentamer ratio or virion infectivity, demonstrating uncoupling of these phenotypes.

**IMPORTANCE:**

The emerging picture of HCMV genetic diversity *in vivo* prompts a reevaluation of how *in vitro*-characterized phenotypes, such as the abundance of different viral envelope glycoproteins, virion infectivity, and tendency toward cell-free or direct cell-to-cell spread, reflect viral characteristics *in vivo*. Laboratory examination of HCMV phenotypes has included a limited sampling of the apparent *in vivo* genetic diversity. A widely held model that directly links cell-free and cell-to-cell spread characteristics to glycoprotein display and virion infectivity also presumes that HCMV is predominantly cell-associated *in vivo*. These have implications for intervention strategies, such as calling into question the therapeutic benefit of neutralizing antibodies. Our finding that spread characteristics can be uncoupled from glycoprotein display and virion infectivity suggests a model that includes both cell-free and cell-associated spread as *bona fide* wild-type phenotypes for different allelic haplotypes. This model would allow a broader examination of neutralizing antibodies as correlates of protection.

## INTRODUCTION

One of the nine human herpesviruses, human cytomegalovirus (HCMV) has an average global prevalence of 83%, although this varies by region and stratifies by age, as the longer an individual lives, the more chances they have to seroconvert (1). Infections are typically mild in immunocompetent individuals but can be dangerous in those with compromised or naive immune systems, including those living with HIV/AIDS, transplant recipients, and newborn infants (2–5). An estimated 1 in 150 infants worldwide are born with HCMV infection, leading to problems ranging from stunted growth to hearing loss and even microcephaly (3). Prior maternal infection does not offer broad protection against subsequent infections or prevent intrauterine infection of developing fetuses (6–8). Consequently, congenital HCMV infection is more common in regions of the world with higher seroprevalence among adults (9). Success of interventions, including vaccines and drug therapeutics, has been limited (10–12), and this may be due in part to an inadequate understanding of how the genetic variation of HCMV impacts viral phenotypes and how *in vitro*-characterized phenotypes relate to *in vivo* viral characteristics.

The scope and dynamics of HCMV genetic variation are becoming more clear (13–23). The majority of nucleotide (*nt*) diversity in the HCMV genome is clustered into discrete regions interspersed among longer stretches of highly conserved sequences. High linkage-disequilibrium (LD) in the variable regions indicates that the genes present are stable, fixed alleles. Low LD in the conserved sequences suggests frequent recombination that would shuffle the variable, allelic genes into a vast number of combinations, or “allelic haplotypes.” The intrahost *nt* diversity of HCMV can be comparable to that of some RNA viruses, likely reflecting mixed haplotype infections rather than rapid genetic drift, and is more consistent with the higher fidelity of DNA replication (24). *In vitro* characterization of HCMV phenotypes has involved a limited sampling of the apparent *in vivo* genetic diversity, and questions about artifactual *in vitro* genetic drift are complicated by a lack of understanding of the genetic correlates of phenotypes and how gross physiologic differences among variants can influence the relative contribution of any specific mechanism to overall observed replication and spread characteristics.

We have reported extensive phenotypic variation among three HCMV BAC clones derived from distinct clinical isolates: TB40-BAC4 (TB), TR-BAC (TR), and Merlin-BAC (ME). These three clones differ in the amounts of two entry-mediating glycoprotein complexes in the virion envelope, gH/gL/gO (“trimer”) and gH/gL/UL128-131 (“pentamer”) (25, 26). The shared gH/gL portion of these complexes contributes to the fusion apparatus, acting as a cofactor for the fusion protein gB, whereas the accessory subunits gO and the UL128-131 subcomplex are receptor-binding domains that influence cell-type-specific tropism (27–30). TB and TR virions contain large amounts of trimer and low levels of pentamer, near the detection threshold of immunoblotting, whereas ME expresses less total gH/gL, the bulk of which is pentamer (25, 26). The ME BAC clone was engineered with tetracycline (tet) operator sequences in the UL131 promoter such that progeny virus produced in cells expressing the tet-repressor protein (TetR) is drastically reduced in pentamer and slightly increased in trimer (25, 31). Trimer binds PDGFRα and facilitates entry to fibroblasts (30). Consistent with this, TB and TR are considerably more infectious than ME on fibroblasts, and the infectivity of ME is greatly increased by propagation in TetR cells (25). Pentamer promotes infection of epithelial cells by binding NRP-2 and OR14I1 (29, 32). While the infectivity of all three clones is lower on epithelial cells, the comparisons between clones are generally similar, indicating that the low levels of pentamer in TB and TR virions are sufficient and that trimer plays an important role in infection of epithelial cells as well as fibroblasts (25, 33). This, coupled with the low (or lack of) PDFGRα expression in these epithelial cells, suggests other trimer receptors (32, 34–36). The relatively high virion-specific infectivity of TB and TR is consistent with the ability of these strains to spread more efficiently via diffusion of progeny released into the culture supernatants (i.e., “cell-free spread”), whereas ME is more restricted to direct cell-to-cell spread, consistent with its low virion infectivity (37).

Phenotypic shifts during *in vitro* serial passage of HCMV have been widely documented, and some have been correlated with changes to the consensus-level viral genome sequence (38–45). Serial passage experiments reported by Murrell et al. (2016) stand out because they involved BAC-cloned HCMV and, thus, could confidently attribute the genetic changes to *de novo* mutation rather than selection of preexisting variants (40). Within five passes on fibroblast cells, the UL128-131 genes of ME acquired disrupting mutations, whereas these loci were unaffected in TR and TB. The UL128-131 genes of ME were more stable during passage on epithelial cells, consistent with the importance of pentamer for infection of this cell type. In an earlier report, Murrell and colleagues identified a G/T single *nt* polymorphism (SNP) in TB relative to ME at a splice site of the UL128 pre-mRNA (46). When the TB residue (T) was engineered into ME, pentamer expression was reduced, and virion infectivity and cell-free spread on fibroblasts increased (46). The role of this UL128 splice site SNP in pentamer levels was confirmed when a substrain containing the “ME-like” residue (G) at this position was selected from the TB40/e mixed-genotype isolate by passage on epithelial cells and shown to contain higher levels of pentamer (38, 47). These observations have contributed to a model in which HCMV is predominantly cell-associated *in vivo* and the appearance of a cell-free spread phenotype *in vitro* is a direct consequence of mutations, including those that reduce expression of the UL128-131 genes and increase virion infectivity (48)

Several observations are inconsistent with the model that links spread characteristics to trimer:pentamer levels and virion infectivity as universal characteristics of HCMV. First, the TR BAC clone is also low in pentamer, such as TB, but harbors the ME-like UL128 splice site residue (G), suggesting that other genetic variations can influence trimer:pentamer levels. Indeed, Zhang et al. demonstrated that in addition to high expression of the UL128-131 proteins, ME is also low in gO expression compared to TR, and this low gO expression might be related to a reduced expression of UL148, an ER resident protein that regulates endoplasmic reticulum-associated degradation (ERAD) of gO (49, 50). Second, the enhanced virion infectivity and cell-free spread of ME caused by TetR repression of pentamer did not come at the expense of highly efficient cell-to-cell spread (37). Third, the cell-to-cell spread of TB was especially poor despite the production of highly infectious intracellular virus (37). This suggests that the respective specializations of TB and ME for cell-free and cell-to-cell spread are not merely reflections of trimer:pentamer levels and virion infectivity. Rather, ME seems capable of a specific mechanism(s) that promotes direct cell-to-cell spread that TB is not. Together, these observations are inconsistent with the model that views cell-free spread as merely a consequence of mutations that reduce the levels of pentamer in relation to trimer and increase virion infectivity. Here, we describe the generation of a set of TB-ME recombinants that uncouple the causal link of trimer:pentamer levels and virion infectivity from spread characteristics.

## RESULTS

### Construction of TB-ME recombinant library

**Fig 1:**
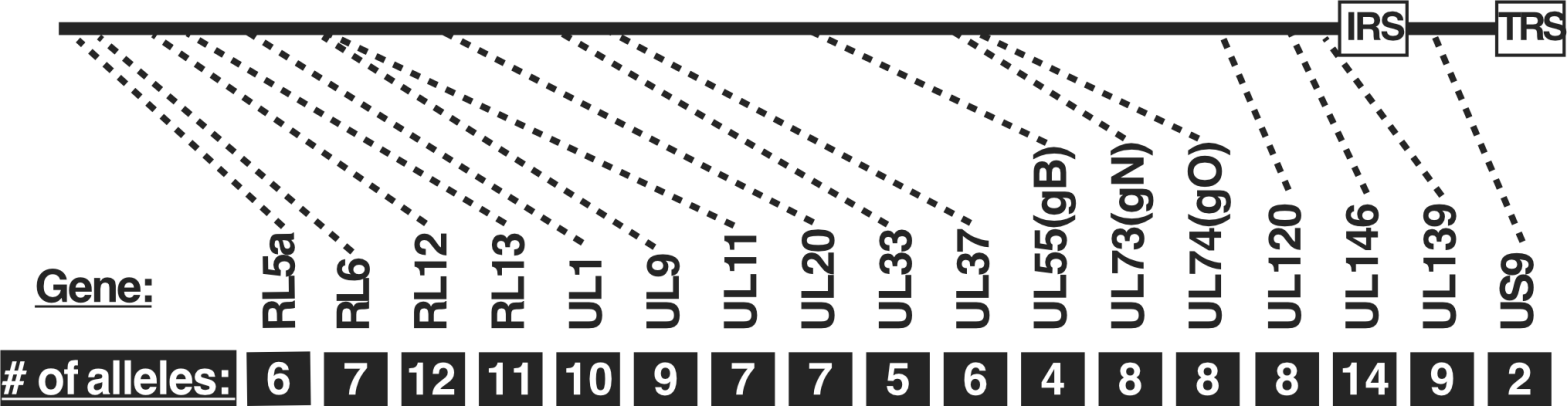
The allelic diversity of HCMV. A diagram of the HCMV genome indicating the approximate location of allelic genes and number of alleles each, based on the analyses of Lassalle and Suarez (17, 20).

Most of the *nt* diversity within the HCMV genome is clustered into discrete regions distributed throughout otherwise highly conserved sequences (20). High LD indicates that the genes in these variability clusters are distinct, fixed alleles. Seventeen of these genes were characterized to have between 2 and 14 alleles (17) (Fig. 1). Table 1 depicts the “allelic relatedness” of the three reference clones—TB, TR, and ME—used in our previous studies. TR shares a different set of four alleles with both TB and ME, whereas the two most phenotypically distinct clones, TB and ME, share only two alleles. To test the genetic linkage between the three phenotypes—(i) trimer:pentamer ratio, (ii) virion infectivity, and (iii) spread mode preference—a set of inter-clone recombinants was generated using TB and ME as the parental strains.

**TABLE 1:** Allelic relatedness of HCMV BAC clones

Clones	# of alleles shared (*genes*)
TB-TR	4 (*UL9, UL33, UL55, US9*)
TR-ME	4 (*RL6, UL11, UL55, US9*)
TB-ME	2 (*UL55, US9*)

Parental TB and ME containing mCherry (mC) or GFP genes in place of US11 (37) were used to coinfect cultures of HFFtet cells (Fig. 1). Note that the parental ME-GFP was propagated on TetR-expressing human foreskin fibroblasts (HFFtet), suppressing the expression of UL131 to enhance the virion infectivity and reduce selection against pentamer (“MT-GFP”) (25, 31). Cells infected by both parental viruses (i.e., GPF+/mC+ cells) were batch-collected by FACS, replated, and maintained for 8 days, at which time progeny virus was harvested from the culture supernatant and infectious units (IU) were determined by fluorescent marker: 1 × 10^6^ (GFP) and 6 × 10^6^ (mC) IU per mL. Since no new uninfected cells were added, 100% of the progeny virus were derived from cells coinfected by TB and ME, and thus, potential recombinants. Moreover, since the GFP and mC marker genes were inserted into the homologous locus in both parental genomes (i.e., US11), no recombinant progeny could harbor both markers without major, and likely highly deleterious, genetic abnormalities. Supernatant virus derived from dual GFP+/mC+ cells was passed through a 0.4 um filter to remove virion aggregates and detached infected cells, and then used to infect HFFtet cells at a low multiplicity to minimize infection by more than one virion per cell. After 2 days, the cells were FACS-sorted to collect single GFP+ and mC+ infectious centers into 96-well plates, along with fresh, uninfected HFFtet cells. The use of HFFtet cells throughout this process reduced selection against any “ME-like” viruses, which are high in pentamer (31).

**Fig 2:**
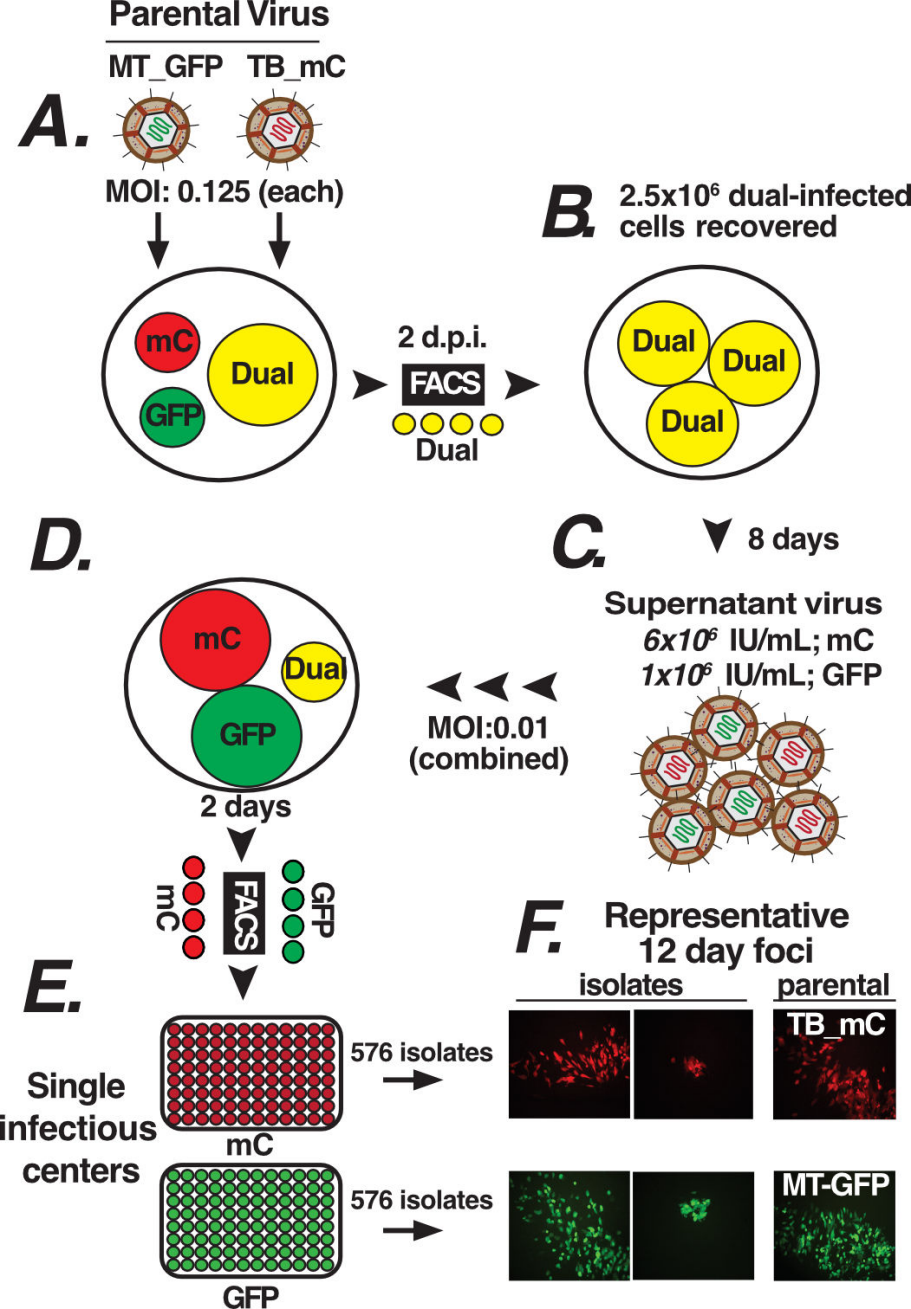
Isolation of virus progeny from TB-ME coinfected cells. (**A**) HFFtet cells were coinfected with parental TB-mCherry (mC) and MT (ME produced in HFFtet cells)-GFP. (**B**) Dual-infected GFP+/mC+ cells were collected by FACS at 2 days post-infection (dpi). (**C**) Progeny virus was purified after 8 days, and GFP and mC infectious units (IU) were determined. (**D**) Fresh HFFtet cells were inoculated at low multiplicity, and (**E**) single GFP+ and mC+ cells were collected at 2 dpi into 96-well plates of HFFtet cells as infectious centers. (**F**) Isolates recovered displayed a range of foci phenotypes.

Of the 1,152 infectious center-derived isolates recovered, 50 were initially selected at random for expansion and characterization, with the remainder stored for future analyses. Focal expansion from infectious centers was monitored visually for 10–20 days. Approximately 70% showed single GFP or mC focal expansion, and these were harvested for analysis. The remaining 30% either had both GFP and mC or showed no focal expansion and were discarded. Whereas both parental viruses formed large, dispersed foci on HFFtet cells, indicative of efficient cell-free spread, the putative recombinant isolates showed a range of focal patterns, from large and dispersed to small and compact (Fig. 2F). Fifteen isolates showing varied focal patterns were selected for expansion and further characterization. Two were subsequently discarded during characterization of recombinant genome structure (see below).

### Restriction fragment length polymorphism (RFLP) analysis revealed complex recombination patterns

**Fig 3:**
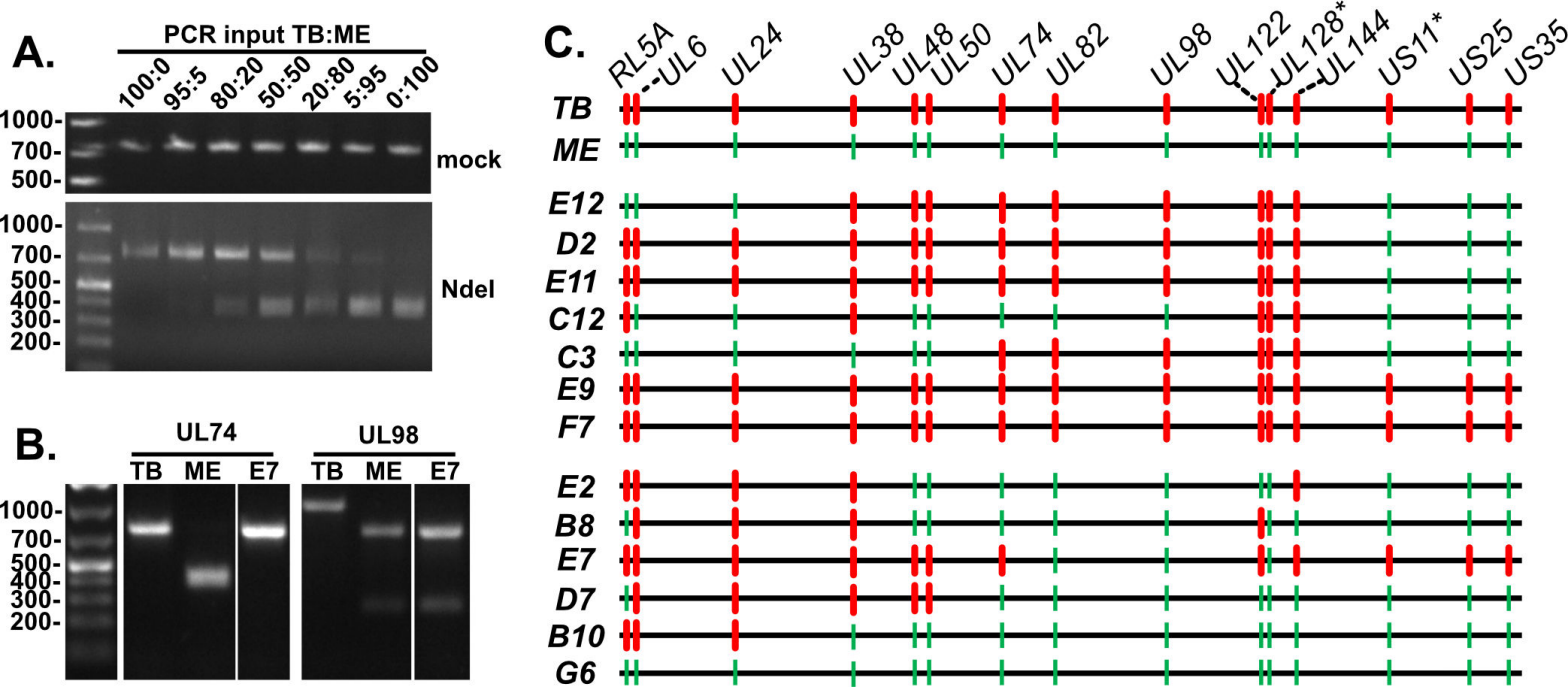
Restriction fragment length polymorphism (RFLP) analysis of recombinant genome structure of TB/ME coinfection progeny isolates. (**A**) TB and ME BAC DNA were mixed in the indicated ratio as input template for PCR reactions with primers targeting UL74 sequences (Table 2). PCR amplicon was mock-digested (top) or digested with Nde I (bottom) and then analyzed by agarose gel electrophoresis and stained with ethidium bromide. (**B**) DNA extracts from cells infected with parental TB, ME, or the isolate E7 were PCR amplified with primers within the UL74 or UL98 loci, then digested with Nde I or Sac I, respectively, and analyzed by agarose gel electrophoresis and stained with ethidium bromide. (**C**) TB versus ME assignments of loci based on RFLP as in Table 2 in three independent experiments. * Assignment of UL128 based on sequencing, not RFLP, and US11 locus based on visible GFP or mC fluorescence.

Recombinant genome structure was assessed by PCR-RFLP analysis. A set of PCR primers was designed with perfect conservation to both parental virus sequences, but sequence polymorphisms near the middle of the PCR amplicon disrupted a restriction nuclease site in one of the two parents (Table 2). Figure 3A shows an example of a predicted PCR amplicon within UL74. The 790 base pair (bp) amplicon from the ME sequence contained an Nde I site near the middle, resulting in fragments of 422 and 368 bp, which comigrated during electrophoresis. T>G and G>A polymorphisms in TB (Table 2) prevented Nde I digestion. In this experiment, the PCR input template was titrated from 100% TB to 100% ME, demonstrating the detection of an approximately 95:5 mixture of parental viruses. Figure 3B shows an example analysis of a TB-ME recombinant isolate, denoted E7, compared to both parental viruses. The UL74 amplicon of E7 was resistant to Nde I, such as the parental TB, whereas the UL98 amplicon was sensitive to Sac I, such as the parental ME. This indicated that isolate E7 was a TB-ME recombinant with at least one recombination crossover point between UL74 and UL98. All 15 isolates were analyzed at 13 loci across the genome, and each locus was assigned as “TB” or “ME” (Fig. 3C). An additional locus, UL128, was assigned as “TB” or “ME” by Sanger sequencing. As mentioned above, two of the 15 isolates were discarded due to discrepancies in assigning TB vs ME of at least one RFLP locus in three independent experiments, suggesting that these isolates were of mixed genotype. Of the remaining 13 isolates, 10 were confirmed recombinant, showing a variety of complex crossover patterns. The RFLP patterns of two isolates matched the parental TB (E9 and F7), and one matched the parental ME (G6). However, given that there were tens of kbp between RFLP loci, it is not possible to conclude that these three isolates were *bona fide* “non-recombinant parental.” There may have been one or more crossover sites between RFLP loci. Indeed, these isolates showed phenotypic differences compared to the parental viruses (see below).

**TABLE 2:** Predicted PCR amplicon sizes and restriction fragment patterns^*a*^

Locus	PCR primers (5′−3′)	Amplicon size	Nuclease	Strain-specific sequence	Fragments
RL5	ACGCGGAAGAGAAACTCACA	TB: 1164	HindIII	TB: CCGT**AAGCTT**CCAA	912, 252
	GACTGGTACATACGCAGGG	ME: 1145		ME: CCGT**cATCcc**ATGT	n.c.^*b*^
UL6	CGTGTAGCGTTTGGCTGTTC	TB: 981	HindIII	TB: GTTT**AAGCTT**AAGT	794, 187
	TCGTCGTGACGATGGTTGTT	ME: 979		ME: GTTT**AAGCcT**AAGT	n.c.
UL24	CGCGAGTCAAACGGGAAGTA	TB: 907	HindIII	TB: CTCC**AAGCTT**TCAC	801,106
	CATCAACGCAGGCCAGGTG	ME: 907		ME: CTCC**AAGCcT**TCAC	n.c.
UL38	CCACGACCACCATCTGTACC	TB: 988	BamH1	TB: TCGC**GGATCC**GCCG	657, 331
	ACTACGACCACGCATAGCAC	ME: 988		ME: TCGC**GGATtC**GCCG	n.c.
UL48	CAAACCCAAGCCTCGCTAAAC	TB: 1071	EcoR1	TB: CAGC**GAgTTC**GAGG	n.c.
	GAAGGTCTGGATGCCGTGTAAC	ME: 1071		ME: TAGC**GAATTC**GAGA	919, 152
UL50	GACCGTGTCTGTCTTGAGCA	TB: 932	HindIII	TB: CCAT**AAGCTT**GCTG	700, 232
	CCATAGGTACTTGACGCGGG	ME: 932		ME: CCAT**gAGCTT**GCTG	n.c.
UL74	GAAAAGACCCATGGAAA	TB: 766	NdeI	TB: ACCA**CAgATa**GGTT	n.c.
	GCTCATGGCGTTAACCAGGTAG	ME: 790		ME: ACCA**CATATG**GGTA	422, 368
UL82	CGTGGGCCAAAGTTGTTGAG	TB: 1104	HindIII	TB: ACCA**AAGCTg**CCGC	n.c.
	TCACCAGCCAGTATCGCATC	ME: 1104		ME: ACCA**AAGCTT**CCGC	854, 250
UL98	TGTCTTACGCCTACCGCATC	TB: 1050	Sac1	TB: GGTC**GAaCTC**TTCC	n.c.
	GGCCGAGACTCGCGTTAATA	ME: 1050		ME: GGTC**GAGCTC**TTCC	780, 270
UL122	TTCGGCCAATTCTGGGAACA	TB: 1110	BamH1	TB: GAAA**aGAcCC**ATGG	n.c.
	ACCCCCTTGGCTTCTTATGC	ME: 1104		ME: GAAA**GGATCC**ATGG	781, 323
UL144	CAGGCTAGAGTATGACGACC	TB: 948	HindIII	TB: CTGA**AAGCTT**TTCC	545, 403
	GGGCGAATAACATGCTGT	ME: 982		ME: CTGA**AAGtTT**TTCC	n.c.
US25	GCTCCGATTTTTCACCGTCG	TB: 1094	BamH1	TB: CCAG**GGgTCC**TGGG	n.c.
	ATCCGACCCGACTATCCACA	ME: 1094		ME: CCAG**GGATCC**TGGG	738, 356
US35	GTGCAACGCGTGAAACAAGA	TB: 1188	HindIII	TB: CAGG**AcGCTT**GGTG	n.c.
	GATGGGTGGTGCTACTCAGG	ME: 1283		ME: CAGG**AAGCTT**GGTG	636, 552

^*a*^ Bold text indicates the nuclease site. Lowercase letters indicate strain-specific nucleotide polymorphism(s) affecting the nuclease site.

^*b*^ “n.c.” indicates not cut.

### Phenotypic analysis of recombinant HCMV

While recombinants were isolated and expanded in HFFtet cells to reduce selection against the UL128-131 locus for “ME-like” viruses, high multiplicity (i.e., single-round infection) “burst” stocks were generated on HFF lacking TetR expression to analyze phenotypes.

### Display of gH/gL/gO (trimer) and gH/gL/UL128-131 (pentamer)

**Fig 4:**
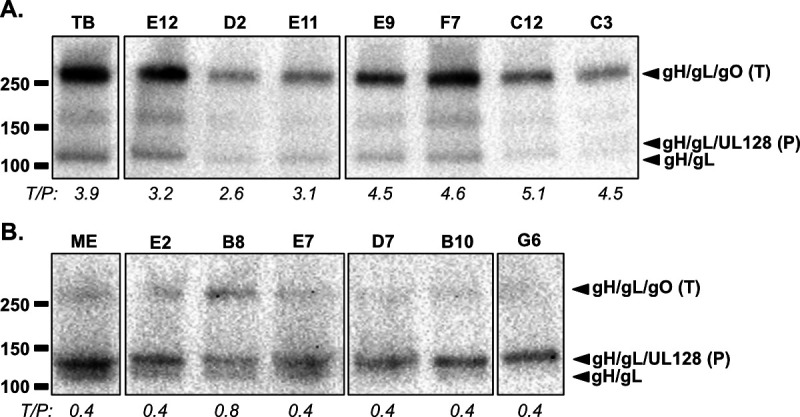
Immunoblot analysis of gH/gL complexes in parental and recombinant HCMV. Virion extracts of TB-like (**A**) or ME-like (**B**) HCMV pelleted from HFF culture supernatants were separated on non-reducing 4–20% gradient SDS-PAGE gels, transferred to nylon membranes, and probed with anti-gL antibodies. Mobility markers indicated in kDa (left). Locations of gH/gL/gO (trimer “T”), gH/gL/UL128 (pentamer “P”), and gH/gL indicated to the right of each panel. Loads were not normalized to number of virions. Ratio of band densities trimer/pentamer (T/P) indicated beneath each lane in italics as determined using FIJI V.2.16/1.54p. All lanes shown were digitally cropped from the same gel images in panels A and B, respectively, and are representative of at least three biological replicates.

The relative abundance of trimer and pentamer (i.e., trimer:pentamer ratio) of the recombinant isolates was analyzed by immunoblot and band densitometry. Consistent with the previous results (26), TB virions contained far more trimer than pentamer, with a ratio of 3.9 in the experiment shown, whereas ME virions contained more pentamer than trimer, with a ratio of 0.4 (Fig. 4A and B). The ratios of most recombinants were either much greater than 1.0, like the parental TB (Fig. 4A), or much less than 1.0, like the parental ME (Fig. 4B). Only one isolate (B8) had a nearly balanced amount of trimer and pentamer with a ratio of 0.8. Given that this ratio was lower than 1.0, isolate B8 was included among the “ME-like” set for further analyses.

### Virion-specific infectivity

**Fig 5:**
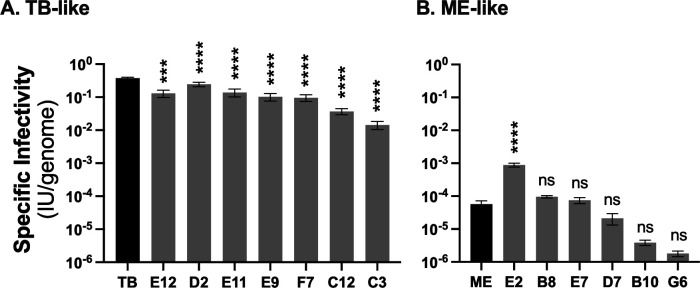
Specific infectivity of parental and recombinant HCMV. Extracellular HCMV stocks were quantified by qPCR for viral genomes and infectious units (IU) determined by flow cytometry quantification of GFP or mC expression in HFF cells 3 days post-infection. Average IU/genome from at least three biological replicates is plotted with error bars representing standard deviation. Asterisks indicate *P* values of differences between recombinants and the parental TB (**A**) or ME (B). *** <0.001, **** <0.0001, ns = not significant, calculated by one-way analysis of variance (ANOVA) with Dunnett’s post hoc test comparing each recombinant to TB (**A**) or ME (**B**).

We previously reported that the per-genome infectivity of TB stocks was log-fold higher than for ME stocks (25, 37, 51). While the specific infectivity of the recombinant isolates corresponded categorically to the trimer:pentamer ratios, with “TB-like” recombinants displaying much higher infectivity than the “ME-like” viruses, there was substantial intragroup variability that could not be explained by small fluctuations in trimer:pentamer ratio (Fig. 5A and B). Among the TB-like recombinants, E12, D2, E11, E9, and F7 were marginally less infectious than the parental TB (1.5- to 4-fold), whereas C12 and C3 were 10.2- and 26.3-fold less infectious, respectively (Fig. 5A). Among the ME-like recombinants, E2 was 15.4-fold more infectious than the parental ME, whereas B8, E7, and D7 were not statistically different from ME. Recombinants B10 and G6 were 14.8- and 31.7-fold less infectious than ME, respectively. However, these differences were not statistically significant, likely due to variance reflecting the lower limits of the infectivity measurement.

### Spread characteristics

**Fig 6:**
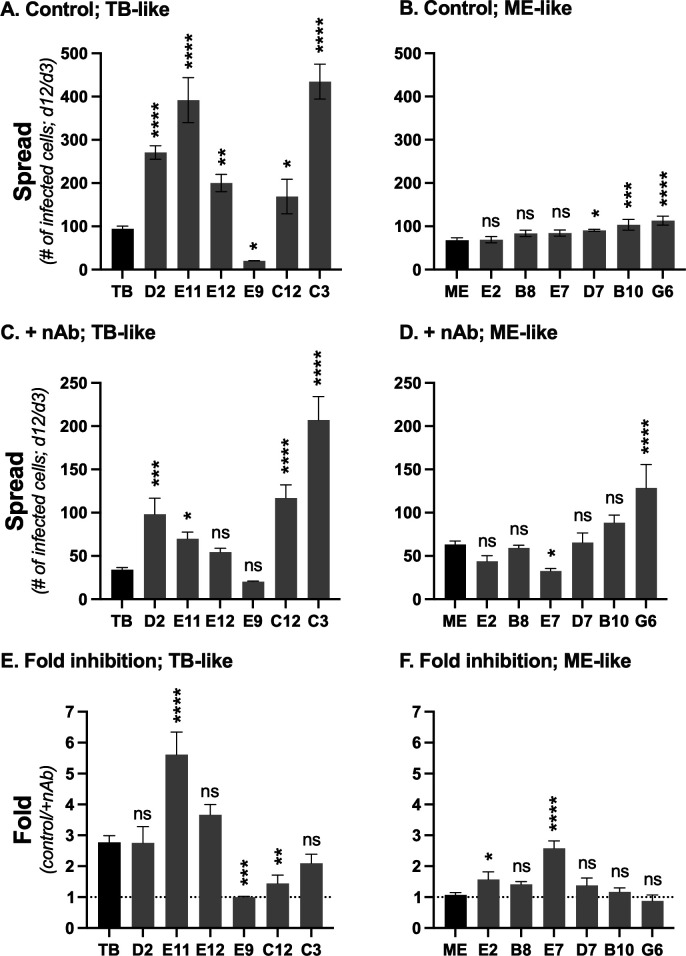
Spread characteristics of parental and recombinant HCMV. (**A and B**) Confluent monolayers of HFF cells were infected at low MOI with the parental (TB or ME) or recombinant isolates. At 3 and 12 days post-infection, the number of infected cells was determined by flow cytometry detection of GFP or mC. Plotted are the average number of infected cells at day 12 per infected cell at day 3, from three biological replicate experiments. Error bars indicate the standard deviation. (**C and D**) As shown in panels A and B, except cultures were maintained in medium supplemented with 50 µg/mL anti-gH mAb 14-4b. (**E and F**) The ratio of values A:C and B:D, with error propagated. Asterisks indicate *P* values of differences between recombinants and the parental TB (**A, C, E**) or ME (**B, D, F**). * ≥0.05, ** <0.01, *** <0.001. **** <0.0001, ns = not significant, calculated by one-way analysis of variance (ANOVA) with Dunnett’s post hoc comparing each recombinant to TB (**A, C, E**) or ME (**B, D**).

Despite the dramatic differences between TB and ME in extracellular virion infectivity, we previously showed that the clones spread through HFF cultures at comparable rates, with TB predominantly via diffusion of highly infectious extracellular virions that could be efficiently blocked by nAb, and ME predominantly via direct cell-to-cell spread that was less sensitive to nAb inhibition (37). There was considerable variation in spread efficiencies among the TB-like recombinants (Fig. 6A). Recombinants D2, E11, E12, C12, and C3 each spread more efficiently than the parental TB (2.8-, 4.2-, 2.1-, 1.8-, and 4.6-fold, respectively), whereas spread of E9 was reduced 4.6-fold. There was less variation among the ME-like recombinants with only D7, B10, and G6 statistically different from the parental ME (1.3-, 1.5-, and 1.7-fold greater, respectively) (Fig. 6B). The reduced spread of E9 compared to parental TB correlated with a difference in specific infectivity (i.e., approximately 4-fold reduced in both specific infectivity and spread). In contrast, none of the enhanced spread could be explained by enhanced virion-specific infectivity, as all these recombinants had decreased infectivity compared to the parental TB or ME (compare Fig. 5A and B with Fig. 6A and B).

In parallel spread experiments, nAb were included in the culture medium to assess the contribution of extracellular virus to spread (i.e., cell-free spread) (Fig. 6C through F). Consistent with the previous results (37), spread of parental TB was strongly inhibited by nAb, indicative of predominantly cell-free spread, whereas spread of ME was not affected by nAb, indicative of predominantly direct cell-to-cell spread. The spread of recombinant E11 was 2-fold more sensitive to nAb than the parental TB, suggesting that the enhanced spread efficiency of this recombinant was due to increased cell-free spread. In contrast, recombinant C12 was 1.9-fold less sensitive to nAb inhibition, consistent with an increased capacity for direct cell-to-cell spread. Spread of recombinant E9, which was 4.6-fold reduced compared to TB (Fig. 6A), was completely unaffected by the presence of nAb (Fig. 6C and E), further suggesting that the reduced spread efficiency was due to decreased infectivity of the extracellular virions, and that the remainder of spread was predominantly via a low-efficiency cell-to-cell mechanism. Among the ME-like recombinants, E2 and E7 spread with comparable efficiency as the parental ME but were more sensitive to nAb inhibition (1.5- and 2.5-fold, respectively), indicating a shift from reliance on direct cell-to-cell spread to more cell-free spread.

## DISCUSSION

The unveiling of the vast and complex nature of HCMV genetic diversity compels a reevaluation of how *in vitro* characterizations of HCMV phenotypes reflect viral characteristics *in vivo*. The tendency of clinical isolates to display a cell-associated phenotype during initial culturing has been viewed to support the notion that HCMV *in vivo* is principally cell-associated and that a cell-free phenotype is an *in vitro* artifact linked to mutations, including those that reduce pentamer expression (48). Indeed, leukocyte depletion has been linked to reduced transmission of HCMV during blood transfusions, arguing against large amounts of infectious, cell-free HCMV in the blood of these individual donors (52, 53). While this is consistent with a model of hematogenous dissemination of HCMV via monocyte/macrophages (54, 55), these were small-scale studies that do not offer broad insights into the roles of cell-free and cell-associated virus in other aspects of HCMV pathogenesis. Moreover, while cell association is a commonly observed phenotype for clinical isolates, cell-free phenotypes have also been reported during initial culturing (18, 42). Comprehensive understanding of the *in vivo* role of phenotypes, such as cell-free and cell-to-cell spread, will benefit from a more complete understanding of the associated genetic signatures.

The HCMV BAC clones TB and ME display opposite extremes *in vitro* phenotypes including the levels of the entry-mediating glycoprotein complexes trimer and pentamer, virion-specific infectivity, and preference for cell-free or cell-to-cell modes of spread (25, 26, 37, 50, 51, 56). Consistent with these phenotypic extremes, TB and ME are mismatched at 15 of the 17 allelic genes. To test the genetic linkage between three phenotypes, (i) trimer:pentamer ratio, (ii) virion infectivity, and (iii) spread mode preference, we generated a set of TB-ME recombinants by isolating progeny from cells coinfected with TB and ME and assessing recombinant genome structure by targeted PCR-RFLP. One limitation of the PCR-RFLP approach was the possibility that stochastic PCR amplification of one of multiple homologous targets over the others in the early cycles could give the appearance of genetic purity when, in fact, the isolate was of mixed genotype. This was addressed with a TB-ME genome titration experiment that demonstrated the ability to detect approximately a 5% minor variant, and a stringent standard was set requiring agreement among three independent experiments across all RFLP loci for each isolate. Indeed, two of the original 15 isolates selected for analysis were discarded due to inconsistent results in RFLP patterns in the three experiments. If a minor variant was present in an isolate below the level we could detect, it is unlikely that this would influence the observed phenotype, since experiments performed with only 5% of the virus input fail to detect trimer or pentamer on immunoblots, virion infectivity, or viral spread. Thus, while the PCR-RFLP may be considered low resolution in not showing the exact locations of crossovers, the approach demonstrated that the isolates were *bona fide* TB-ME recombinants with a variety of complex crossover patterns.

Although we cannot rule out *de novo* mutations as contributing to the observed phenotypic differences among recombinants, this seems less likely than mixing of parental virus polymorphic genes via recombination. As noted, while the notion that HCMV rapidly acquires widespread *de novo* mutation during *in vitro* propagation in fibroblasts is commonly held, few studies have been able to discriminate *de novo* mutations from selection among multiple preexisting genetic variants by beginning with genetically pure BAC clones. Studies that have tracked genetic changes during passage of pure BAC clones have documented remarkable stability, with localized mutations observed for some clones but not others (40, 57). Indeed, the degree and nature of phenotypic diversification observed among the TB-ME recombinants far exceeds any we have observed with either parental TB or ME in isolation.

The first major phenotypic discrepancy between TB and ME that we reported was that TB virions contained far more trimer than pentamer, whereas ME virions contained more pentamer (26). The genetic basis of this difference appears to be complex. A G/T polymorphism in TB relative to ME was shown to reduce the splicing efficiency of the TB UL128 pre-mRNA, and when the TB residue (T) was engineered into ME, the assembly and expression of pentamer into ME virions were reduced (40, 46). More recently, the clinical isolate TB40/E (the source of the TB40-BAC4 clone that we abbreviate as “TB”) was shown to contain both variants, and the effect of the G/T polymorphism on UL128 expression was corroborated (47). Our results align with these reports, as all but one of the TB-ME recombinants analyzed retained either a “TB-like” or “ME-like” trimer:pentamer ratio, and this corresponded with the presence of the TB or ME UL128 sequence. However, UL128 polymorphism is unlikely to be the sole factor dictating trimer:pentamer levels since: (i) TetR repression of pentamer expression in ME does not result in a compensating increase in virion levels of trimer (25), and (ii) the TR BAC clone is like ME at this UL128 intron splice site, but is trimer-high/pentamer-low like TB (25). Zhang et al. reported that in addition to high expression of the UL128-131 genes, ME is particularly low in gO expression, and this might be due to low expression of UL148, which can protect gO from ERAD (49, 50). This may help explain why the trimer:pentamer ratios among the TB-like recombinants were more variable than among the ME-like recombinants, i.e., there was a greater dynamic range of gO expression. More recently, Weiler et al. suggested that both the TB and ME trimer:pentamer ratio extreme phenotypes might be anomalous based on their observation that 8/8 recent clinical isolates analyzed had balanced trimer:pentamer ratios (58). Indeed, one of our recombinants (B8) showed a nearly balanced trimer:pentamer ratio, suggesting that it is possible for a single HCMV genome to contain the right collection of genetic polymorphisms to epistatically result in a balanced trimer:pentamer ratio. However, it still may be premature to suggest that a balanced trimer:pentamer ratio is more representative of *in vivo* HCMV than the extremes of TB and ME. Weiler et al. did not provide data to address the possibility that the isolates were genetic mixtures of TB- and ME-like variants, which would be expected to yield balanced trimer:pentamer ratios on immunoblots. Moreover, the clinical isolates analyzed were all collected from the same location within a relatively short period of time, decades after the TB and ME isolates were collected in distinct locations (42, 59). Our observations with TB:ME recombinants support the idea that the trimer:pentamer ratio of HCMV is controlled epistatically by variation in multiple loci across the genome and that whether a clinical isolate presents with balanced or imbalanced trimer:pentamer ratio may be due to the dominance of specific haplotypes circulating in that geographic location and time.

The mechanism(s) linking the extreme disparity in trimer:pentamer ratio between TB and ME to their extreme difference in specific infectivity is unclear. TB virions are highly infectious, whereas the infectivity of ME approaches the lower limits of our infectivity assay (25, 37, 56). The ME clone used in our studies contains Tet operator sequences in the UL131 promoter, such that propagation in TetR-expressing fibroblasts results in virions with dramatically less pentamer and slightly more trimer (denoted as “MT”) (25, 31, 46). Whether the enhanced extracellular infectivity of MT virions compared to ME virions was the result of slightly more trimer or the dramatic reduction in pentamer was not clear. Note that while attachment and entry events are critical to infectivity, the readout of infectivity in these experiments was viral gene expression—many steps downstream of virus entry. A recent study provided evidence that the pentamer complex can induce cellular signaling pathways during entry into fibroblasts that can subsequently inhibit immediate-early (IE) gene expression, thus reducing apparent infectivity (60). This idea is consistent with our observation that the specific infectivity of the TB:ME recombinants grouped categorically with the “TB- or ME-like” trimer:pentamer ratio. Moreover, this offers a plausible mechanism driving the apparent selection against the UL128-131 genes of the pentamer-rich ME clone during passage in fibroblasts and the apparent lack of selection against these genes in the pentamer-low TB and TR clones (40). The 15.4-fold enhanced infectivity of recombinant E2 compared to parental ME indicates that a pentamer-induced inhibition might be at least partially compensated by increases in other mechanisms leading to viral gene expression, such as nuclear docking, uncoating, or subversion of cellular antiviral state mechanisms. Conversely, all of the TB-like recombinants were less infectious than the parental TB (1.5- to 26.3-fold), suggesting that the recombinatorial mixing of TB and ME genes can reduce the efficiency of one or more mechanisms during the establishment of infection, consistent with the observation that the TB clone is highly specialized to cell-free spread (37). Together, these observations highlight the complex, multi-mechanism nature of “infectivity,” using the viral gene expression as the readout.

In our usage, the phenotype “spread” represents the totality of mechanisms involved in the viral replication cycle over multiple generations, and again, TB and ME show dramatic differences. TB and ME spread at similar rates in fibroblast cells (i.e., the number of new infected cells per initially infected cell, over time), but while spread of TB relies predominantly on progeny virions released to the culture supernatant that can be efficiently blocked by nAb (i.e., “cell-free” spread), ME predominantly spreads directly cell-to-cell, a process that is much less sensitive to nAb inhibition (37). There is a logical connection between the production of highly infectious extracellular virions and efficient cell-free spread, but the mechanistic basis of efficient cell-to-cell spread is more complicated. While repression of pentamer during ME replication dramatically improves extracellular virion infectivity, this does not come at the expense of efficient cell-to-cell spread (37). Moreover, the intracellular virions of TB are far more infectious than those of ME when liberated from the infected cells by sonication, yet cell-to-cell spread of TB is comparatively inefficient (37). Together, these observations indicate that some aspect(s) of ME physiology allows efficient cell-to-cell spread despite poorly infectious progeny virions, whereas TB is deficient, despite producing highly infectious intracellular virions. In the experiments reported here, there was more variability in spread characteristics among the TB-like recombinants than among the ME-like set. The increased spread of recombinants D2, E11, E12, and C3 compared to parental TB was accompanied by either equal or greater sensitivity to nAb inhibition, suggesting predominant cell-free spread. However, this is counterintuitive to the observation that these recombinants were reduced in specific infectivity compared to TB. Thus, it may be that the mixing of TB and ME genes in these recombinants resulted in an enhancement of some other mechanism associated with cell-free spread, such as the production and release of greater quantities of progeny into the culture supernatant. In contrast, the spread of isolate E9 was reduced relative to TB, which might be explained by this isolate’s reduced specific infectivity. The spread of recombinant C12 was both more efficient and less sensitive to nAb inhibition than TB, suggesting the acquisition of ME genes that allowed for more efficient cell-to-cell spread despite maintaining a high trimer:pentamer ratio. Among the ME-like recombinants, E2 and E7 stand out as spreading comparably to ME, but with greater sensitivity to nAb inhibition. For E2, this was consistent with an increased specific infectivity of extracellular virions, suggesting a shift toward a greater contribution of cell-free spread despite a low trimer:pentamer ratio. However, the increased nAb inhibition of E7 could not be explained by infectivity. Thus, we observed several recombinants that uncoupled spread characteristics from timer:penamer ratio and virion infectivity, contrary to the model that links these phenotypes.

The regions of the HCMV genome between the allelic genes show strong sequence conservation and recombination signatures (20, 21). Questions remain as to whether these recombination signatures represent rare events accumulated over the evolutionary history of HCMV or an ongoing, dynamic process influencing HCMV pathogenesis, as well as intervention successes and failures. Our results suggest that recombination is a common outcome when a single cell is infected by two genetically distinct HCMV variants. This is consistent with the model of recombination-dependent replication, which posits that efficient DNA replication of herpesviruses requires recombination to cope with single- and double-strand DNA breaks (61). While the RFLP analyses demonstrated varied and complex recombination patterns, the resolution was not sufficient to allow identification of candidate genes and epistatic relationships influencing the observed phenotypic variation. We had previously reported that mixing of gH and gO alleles can epistatically impact the efficiency of fusion regulated by trimer binding to the receptor PDGFRα and Ab neutralization on conserved anti-gH epitopes (56, 62). The short intergenic region between UL74 (gO) and UL75 (gH) shows relatively low LD (20), suggesting that crossovers can occur. This offers a potential explanation for differences among recombinants in specific infectivity and inhibition of spread by nAb. Among the recombinants, only E7 had an RFLP pattern consistent with a recombination mixing the TB and ME alleles of gH and gO, since the UL74 matched TB and the UL82 matched ME. However, given the thousands to tens of thousands of Kbp between RFLP loci, crossover points could not be identified, and *de novo* acquired point mutations could not be excluded.

Given the dramatic phenotypic differences among the few strains and BAC clones that have been studied in the laboratory, and the vast genetic diversity of HCMV *in vivo*, the phenotypic variation observed among our set of TB-ME recombinants suggests that the characteristics of HCMV *in vivo* might be dramatically wider, and *in vitro*-characterized phenotypes considered artifactual may be more physiologically relevant than currently appreciated. These have important implications for intervention approaches. Live-attenuated (63–65) and adjuvanted-subunit (66, 67) vaccine candidates have failed to exceed 50% efficacy, and nAb are one of the main immune correlates assessed. The model that views cell-free spread of HCMV as an artifact of *in vitro*-acquired mutations will tend to interpret limited correlation of humoral immunity with protection as evidence that HCMV is exclusively cell-associated *in vivo* (68). Some studies provide evidence that nAbs against the gH/gL glycoprotein complexes can offer protection against transplacental transmission and reactivation in transplant recipients (69–71), whereas others do not (72). A model that includes both cell-free and cell-associated spread as *bona fide* wild-type phenotypes for different allelic haplotypes suggests potential explanations for the limited protection of nAb beyond the cell-associated nature of HCMV. For example, we and others have shown that allelic epistasis between gO and gH can influence the neutralization potency of anti-gH antibodies (56, 73). Ultimately, a broader view of wild-type HCMV that is more consistent with the apparent genetic diversity may greatly improve intervention development.

## MATERIALS AND METHODS

### Cell lines

Primary human foreskin fibroblast cells (HFF; Thermo Fisher Scientific) and tetracycline repressor protein (TetR)-expressing HFF (HFFtet) (37) were cultured in Dulbecco’s modified Eagle’s medium (DMEM, Sigma) supplemented with a mixture of 5% heat-inactivated fetal bovine serum (FBS, Rocky Mountain Biologicals, Missoula, MT, USA), 5% Fetalgro (Rocky Mountain Biologicals, Missoula, MT, USA), penicillin-streptomycin, gentamicin, and amphotericin B.

### Human cytomegaloviruses (HCMV)

BAC clone TB40/e-BAC4 (TB) was provided by Christian Sinzger (University of Ulm, Germany) (74). BAC clone Merlin (pAL1393), which contains tetracycline operator sequences within the transcriptional promoter of UL131, was provided by Richard Stanton (Cardiff University, United Kingdom) (31). BAC clones were modified to express green fluorescent protein (GFP) or the monomeric red fluorescent protein, mCherry (mC), using en passant recombineering to replace the US11 gene with the eGFP or mC gene (37). Infectious HCMV was recovered by electroporation of BAC DNA into MRC5 cells (33), which were then co-cultured with either HFF cells (TB) or HFFtet cells (ME). Supernatant virus was harvested, and IU were determined by flow cytometry (37). ME stocks propagated in HFFtet cells were denoted “MT.”

### TB-ME recombinant isolates

Six confluent 150 × 20 mm tissue culture plates of HFFtet cells were inoculated with 7.6 × 10^6^ IUs (MOI = 0.25 IU/cell), combined from TBus11mCherry (TB_mC) and MTus11GFP (MT_GFP), diluted to a total volume of 10 mL per plate, and incubated at 37°C for 4 h. Inoculum was aspirated and replaced with DMEM + 2% FBS. The infections were incubated for 2 days before the cells were lifted with 0.5× trypsin and pelleted at 500 g for 5 min. Half of the cells were resuspended in 1 mL sorting buffer (F-12/DMEM; Sigma + 10% FBS), and the other half were resuspended in 1 mL DMEM + 2% FBS and frozen at −80°C. F-12/DMEM was used as the sorting buffer because it is HEPES-buffered to maintain pH in ambient CO_2_ during sorting, which lasted approximately 3 h. Cells were sorted using a BD Aria Fusion flow-activated cell sorter (FACS). Cells were gated from debris using forward scatter-area and side scatter-area, and single cells were gated using forward scatter-width and forward scatter-height. GFP+ and mC+ gates were drawn using non-tagged virus-infected cells as negative controls. Approximately 2.5 × 10^6^ dual GFP+/mC+ were collected, representing about 40% of the total infected cells, and plated on a single 100 × 20 mm tissue culture plate. The survival rate was about 75% after 3 days. At 8 days post-sorting (10 days post-infection), supernatant was harvested and frozen at −80°C in 1 mL aliquots. IUs were determined as above: mC: 6 × 10^6^ IU/mL; GFP: 1 × 10^6^ IU/mL.

To isolate individual progeny viruses, two 100 × 20 mm plates of confluent HFFtet cells were inoculated with supernatant (0.4 µM filtered to remove cells, cellular debris, and viral aggregates) in 5 mL/plate total volume (MOI of 0.009 and 0.18 IU/cell) for 4 h at 37°C. The inoculum was then replaced with 10 mL of DMEM + 2% FBS. Two MOIs were used to decrease the probability of coinfections while ensuring enough infected cells for FACS collection. At 3 days post-inoculation, the cells were lifted with 0.5× trypsin from both and resuspended together in 1 mL sorting buffer. Simultaneously, twelve 96-well plates were seeded with HFFtet cells at a density that would result in about 125% confluency upon adherence (planning for an average 75% survival rate). The infected cells were then sorted using stringent gating for only GFP+ or only mC+ cells, and one infected cell was deposited per well of the 96-well plates (six plates of GFP+ cells and six plates of mC+ cells). Beginning at day 10 post-sorting, wells were examined by fluorescence microscopy for focus formation. Wells with no foci or with both GFP and mC were discarded. Wells with only GFP or mC were harvested by scraping cells into the supernatant with a cut pipet tip, and then, the cells and supernatant together were stored at −80°C. To amplify an isolate, the entire sample (supernatant and cells) was plated on one well of a 48-well dish along with fresh HFFtet cells at a density to achieve confluence. Most isolates were expanded after 10 days, although some slower-spreading isolates were allowed to spread for an additional 10 days prior to expansion. In an attempt to limit selection of cell-free or cell-associated viruses, expansion involved collecting the culture supernatant and intact cells together and transferring them to a 6-well dish along with fresh HFFtet. For experiments, high MOI “burst” supernatant stocks were generated on HFF (non-TetR-expressing) as described previously (25).

### Restriction fragment length polymorphism (RFLP) genotyping

DNA was extracted from infected cells using the PureLink genomic DNA minikit (Thermo) and PCR amplified (Platinum SuperFi II DNA Polymerase; Thermo) with primers listed in Table 2. PCR amplicons were then digested with the indicated restriction enzymes (NEB BioLabs) and analyzed on 1% agarose gels. Purified BAC DNA was used in experiments involving the titration of parental TB and ME as PCR inputs.

### Immunoblotting

Supernatant virions were analyzed by immunoblotting as described previously (50). Briefly, virions were solubilized in 2% SDS–20 mM Tris-buffered saline (TBS) (pH 6.8), separated on 4–20% gradient SDS-PAGE (BioRad) under nonreducing conditions, and transferred to polyvinylidene difluoride (PVDF) membranes (BioRad) in 10 mM NaHCO_3_ and 3 mM Na_2_CO_3_ (pH 9.9) containing 20% methanol. Transferred proteins were detected with rabbit anti-gL (75) and anti-rabbit horseradish peroxidase (HRP)-conjugated secondary antibody (Sigma). Following exposure to Pierce ECL Western blotting substrate (Sigma), chemiluminescence was detected using a Bio-Rad ChemiDoc MP imaging system.

### Quantitative PCR

Viral genomes were determined as described previously (25). Briefly, cell-free HCMV stocks were treated with DNase I before extraction of viral genomic DNA (PureLink viral RNA/DNA minikit; Life Technologies/Thermo Fisher Scientific). Primers specific for sequences within UL83 were used with the MyiQ real-time PCR detection system (Bio-Rad).

### Virus spread

As a modification of the method described by Schultz (37), HFF cells were seeded in two 6-well tissue culture plates per sample, at approximately 200,000 cells per well, and allowed to grow to confluence. At 3 days post-confluence, cells were inoculated with about 1,000 IU/well of virus stock in 1 mL DMEM + 2% FBS and incubated at 37°C for 4 h. Inoculum was then replaced with 3 mL per well of DMEM+ 2% FBS or 3 mL DMEM+ 2% FBS supplemented with 50 µg/mL anti-gH 14-4b monoclonal antibody. One 6-well plate was harvested at 3 days post-infection by trypsinizing and fixing cells in 4% formaldehyde in PBS. The second plate was harvested at 12 days post-infection in the same manner. Fixed cells were pelleted, washed, and re-suspended in 1 mL PBS. Infected cells were quantified by flow cytometry.

## Data Availability

All data supporting the findings of this study are presented within the article. Further details on the procedure of all experiments can be obtained from the corresponding author.

## AFTER EPUB

*[This article was published on 30 December 2025 with information missing from Acknowledgments. The Acknowledgments were updated on 7 January 2026 and 17 February 2026.]*
